# The Protective Effect of Aged Garlic Extract on Nonsteroidal Anti-Inflammatory Drug-Induced Gastric Inflammations in Male Albino Rats

**DOI:** 10.1155/2014/759642

**Published:** 2014-04-30

**Authors:** Gehan Moustafa Badr, Jawaher Abdulaziz AL-Mulhim

**Affiliations:** ^1^Biological Sciences Department, Faculty of Science, King Faisal University, P.O. 380, Al Ahsa 31982, Saudi Arabia; ^2^Zoology Department, Faculty of Science, Ain Shams University, Egypt

## Abstract

Natural products have long gained wide acceptance among the public and scientific community in the gastrointestinal ulcerative field. The present study explore the potential effects of aged garlic extract (AGE) on indomethacin-(IN-) induced gastric inflammation in male rats. Animals were divided into six groups (*n* = 8) control group, IN-induced gastric inflammation group via oral single dose (30 mg/kg to fasted rats) two AGE orally administered groups (100 and 200 mg/kg for 30 consecutive days) two AGE orally administered groups to rats pretreated with IN at the same aforementioned doses. The results declared the more potent effect of the higher AGE dose (200 mg/kg) as compared to that of the 100 mg/kg dose in the gastroprotective effects reflected by significant gastric mucosal healing of damage and reduction in the total microbial induced due to indomethacin administration. In addition to the significant effect to normalize the significant increase in malondialdehyde (MDA), myeloperoxidase (MPO), tumor necrosis factor-**α** (TNF-**α**) values, and the significant decrease in the total glutathione (tGSH), superoxide dismutase (SOD), and catalase (CAT) values induced by indomethacin. The results support AGE antioxidant, anti-inflammatory, and antimicrobial potency reflected by the healing of the gastric tissue damage induced by indomethacin.

## 1. Introduction

The side effects of the anti-inflammatory drugs are one of the major problems in developing medicine today. Therefore, the concept of nutraceuticals evolved. Nutraceuticals are medicinal foods that have a role in maintaining wellbeing, enhancing health, modulating immunity, and thereby preventing as well as treating specific diseases [1]. Plants of the genus* Allium* known for their production of steroid, saponins, and organosulfur compounds, such as alliin, ajoene, are representative chemicals. Recently, fresh garlic found to have some interesting biological and pharmacological activities including antifungal and antibacterial effects [2, 3]. Aqueous garlic extract exerts antioxidant action by scavenging reactive oxygen species enhancing cellular antioxidant enzymes superoxide dismutase, catalase, and glutathione peroxidase [4]. Garlic represents an important source of antioxidant phytochemicals such as diallyl sulfide, S-allylmercaptocysteine, and ajoene, which is the optimal assurance for neutralizing free radical-mediated inflammation. It possesses hepatoprotective, neuroprotective, genoprotective, immunoprotective, and antioxidative activities [5–9].

Nonsteroidal anti-inflammatory drugs (NSAIDs) are widely used in the treatment of fever, pain, and inflammation. However, these drugs have some side effects, especially on the gastrointestinal tract such as gastric mucosal erosions, ulcerations, bleeding, and perforations. Many studies suggested that the mechanisms for the gastric damage caused by NSAIDs are inhibition of prostaglandin synthesis and inhibition of epithelial cell proliferation in the ulcer margin [10–12]. They stimulate HCl secretion and cause weakness of mucous gel layer, which act as barrier by decreasing mucin production and increasing the secretion of bicarbonate from gastric and duodenal mucosa [13]. Indomethacin (IN) is a nonsteroidal anti-inflammatory drug commonly used to reduce fever, pain, stiffness, and swelling. It works by inhibiting the production of prostaglandins, which normally protect the gastrointestinal mucosa from damage by maintaining blood flow and increasing mucosal secretion of mucous and bicarbonate [14]. Indomethacin has been shown to produce sever gastric damage in rats than did others and has become the preferred drug for inducing ulcer models [11, 12, 15]. The increased levels of reactive oxygen species (ROS) reported in the mechanism of both stress and IN-induced gastric damage [16]. The important roles of oxygen reactive species (ROS) which cause lipid peroxidation (LPO) have been known to play a critical role in the development of pathogenesis in acute gastric damage induced by stress, ethanol, and NSAIDs [17, 18].

Therefore, the present study was designed to explore the potential anti-inflammatory effects of AGE on an IN-induced gastric inflammation in male albino rats and to evaluate its effects on antioxidant parameters in rat stomach tissue and the level of TNF-*α*, a cytokine, plays an important role in inflammation.

## 2. Material and Methods

*Experimental Animals.* The present study was carried on (48) adult male albino rats (*Rattus norvegicus*), weighing (200 ± 20 g). Animals were housed in environmentally controlled conditions (temperature of 22 ± 2°C) with a 12 h light/dark cycle and had free access to commercial rodent pellets and water* ad libitum* in accordance with the National Institutes of Health Guide for Care and Use of Laboratory Animals [19].

*Chemicals.* Aged garlic extract (AGE) was purchased as capsules from Wakunaga of America CO., LTD (Mission Viejo, CA, USA). Indomethacin (IND) was purchased from Sigma Chemical Co., St. Louis, MO, USA.

*The Experimental Design.* The animals were randomly divided into six groups (*n* = 8) and subjected orally via stomach tube daily for 30 consecutive days:

1. * control* subjected water.
2. * indomethacin-induced gastric inflammation (IN)*: the animals were deprived of food but had free access to tap water 24 h before ulcer induction with a single oral dose of indomethacin 30 mg/kg.
3. * aged garlic extract (AGE 100)*: administrated AGE (100 mg/kg).
4. * aged garlic extract (AGE 200)*: administrated AGE (200 mg/kg).
5. * indomethacin + aged garlic extract (IN + AGE 100)*: administrated AGE (100 mg/kg) six hours after IN treatment.
6. * indomethacin + aged garlic extract (IN + AGE 200)*: administrated AGE (100 mg/kg) six hours after IN treatment.

*Tissue Sampling.* At the end of the experimental duration, the animals were sacrificed (4 h after indomethacin administration in IN-group). Immediately blood collected for serum preparation and the stomachs was separated out the body.

*Macroscopic and Histopathological Studies*. The isolated stomachs from the control and treated groups were cut along the greater curvature and washed in ice-cold saline and spread out with pins on a cork board and then photographed using a digital camera to assess the inflamed areas of the mucosa in all tested groups. The total gastric mucosal erosive lesions were measured (mm^2^) with a dissecting microscope under ×20 magnification [20].

The stomachs were then divided into three parts; the first part was fixed in 10% formalin for histopathological examination. Paraffin sections 5 *μ*m in thickness were prepared and stained with Haematoxylin and Eosin stain (H&E) to verify histological details [21].

*Total Gastric Microflora. *Stomach content of the second part rinsed using 10 mL NaCl 0.9%, collected in sterile tube and diluted by buffered sodium chloride peptone solution pH 7. Samples filtered using membrane filtration method, transferred one of the membranes on Casein soybean digest agar plate with sterile forceps, and were incubated 5 days at 30°C–35°C for bacteria. While, the other membrane transferred to sabouraud dextrose agar plate, and incubated for 5 days at 20°C–25°C for fungi. The test strains were* Candida albicans* (ATCC 2091) and* Escherichia coli* (ATCC 8739) (ATCC: American Type Culture Collection, Rockville, MD, USA). The total viable aerobic count is the sum of the bacterial average number of colony-forming units (CFU) found on Casein soybean digest agar [22] and that of fungal on Sabouraud dextrose agar [23].

*Biochemical Investigations.* Serum was used to determine the tumor necrosis factor-*α* (TNF-*α*) using Rat TNF-*α* ELISA (enzyme-Linked Immunosorbent Assay) kit [24] and the third part of stomach tissue was homogenized in a 50 mmol/L phosphate saline buffer (PBS) pH 7.2 under cold condition, using Glass-Teflon homogenizing tube. The homogenate centrifuged at 2500 r/min for 10 min and the supernatant used for the determination of malodialdehyde (MDA) level by the thiobarbituric acid test [25], total glutathione (tGSH) [26], and the enzymes activities of Superoxide Dismutase (SOD) [27], Catalase (CAT) [28], and Myeloperoxidase (MPO) [29].

*Statistical Analysis.* Data was expressed as means ± SE. Statistical analysis was evaluated by one-way ANOVA. Once a significant *F* test was obtained, LSD comparisons were performed to assess the significance of differences among various treatment groups. Statistical Processor System Support “SPSS” for Windows software, Release 20.0 (SPSS, Chicago, IL), was used.

## 3. Results

### 3.1. Effect of AGE on Gastric Inflammation

**Figure 1:**
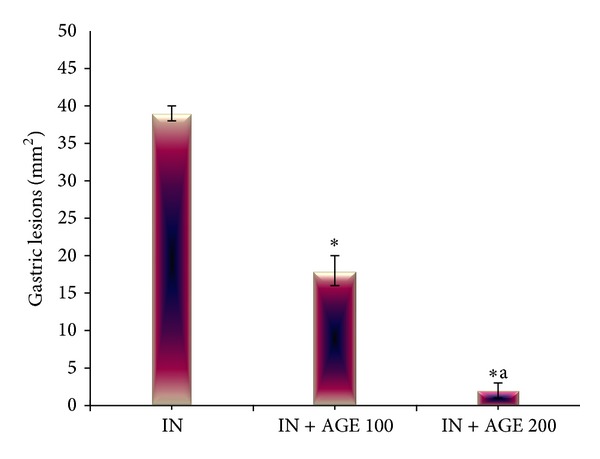
Effect of aged garlic extract (100 or 200 mg/kg) treatments on gastric lesions in stomach from rats treated with indomethacin. Data expressed as mean ± SE (*n* = 8). Significance (*P* > 0.05) between groups represented by superscripts as (∗), significant as compared to control and (a), significant as compared to IN + AGE 100 group.

**Figure 2:**
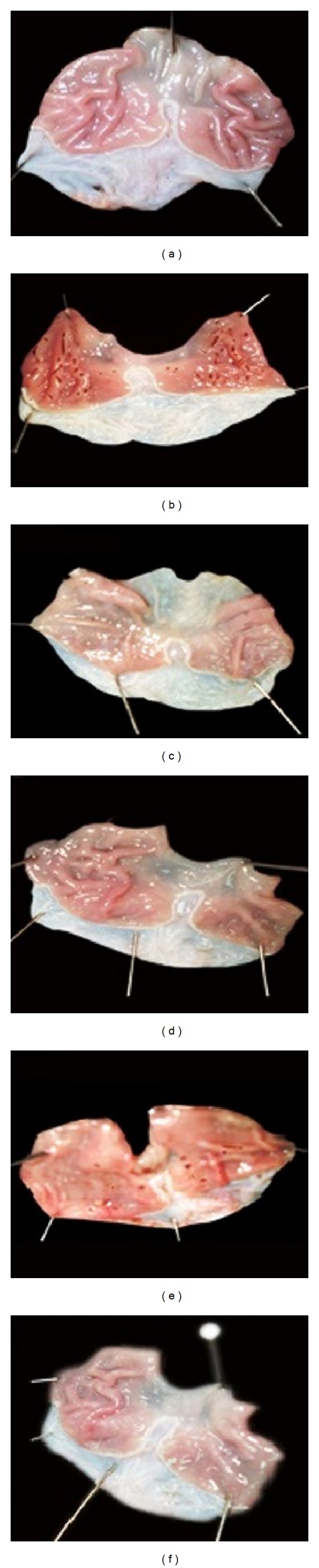
Macroscopic appearance of the gastric mucosa in control group (a), indomethacin treated group (IN) (b), aged garlic extract groups, AGE 100 (c), and AGE 200 (d) and indomethacin + Aged garlic extract groups, IN+AGE 100 (e), and IN+AGE 200 (f).

Indomethacin administration to fasted rats induced gross linear hemorrhagic mucosal lesions. Treatment with AGE showed significant healing effect of the gastric lesions induced by indomethacin in a dose-dependent manner (Figures 1 and 2).

### 3.2. Effect of AGE Total Gastric Microflora

**Figure 3:**
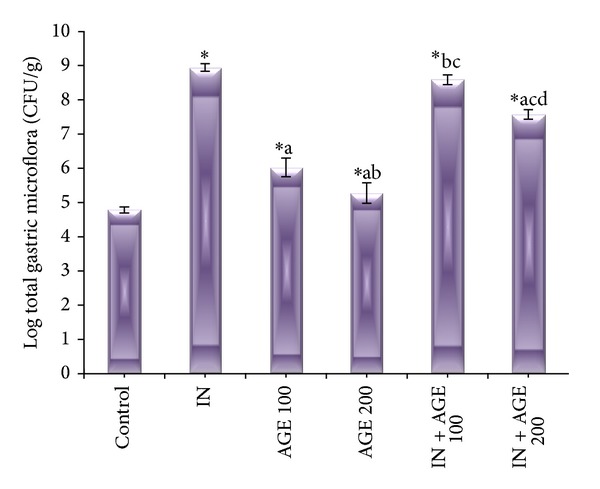
Effect of aged garlic extract (100 or 200 mg/kg) treatments on total gastric microflora in stomach from rats treated with or without indomethacin. Data expressed as mean ± SE (*n* = 8). Significance (*P* > 0.05) between groups represented by superscripts as (∗) significant as compared to control. (a) Significant as compared to IN-group. (b)Significant as compared to AGE 100 group. (c) Significant as compared to AGE 200 group. (d) Significant as compared to IN + AGE 100 group.

Indomethacin induced significant increase in the total microbial count (9.01 ± 0.11 log CFU/g) as compared to that of control (4.78 ± 0.09 log CFU/g). the total microbial count also increased significantly as compared to control in AGE 100 and AGE200 recording (6.04 ± 0.27 log CFU/g) and (5.30 ± 0.03 log CFU/g), respectively, and significantly decreased compared to the IN group result. Treatment with AGE to indomethacin ulcerated group showed significant reduction in the total microbial count in a dose-related effect as compared to IN group value (9.01 ± 0.11 log CFU/g) recording (8.61 ± 0.14 log CFU/g) and (7.61 ± 0.14 log CFU/g) in IN + AGE 100 and IN + AGE 200, respectively (Figure 3).

### 3.3. Histopathological Effect of AGE on Gastric Inflammation

**Figure 4:**
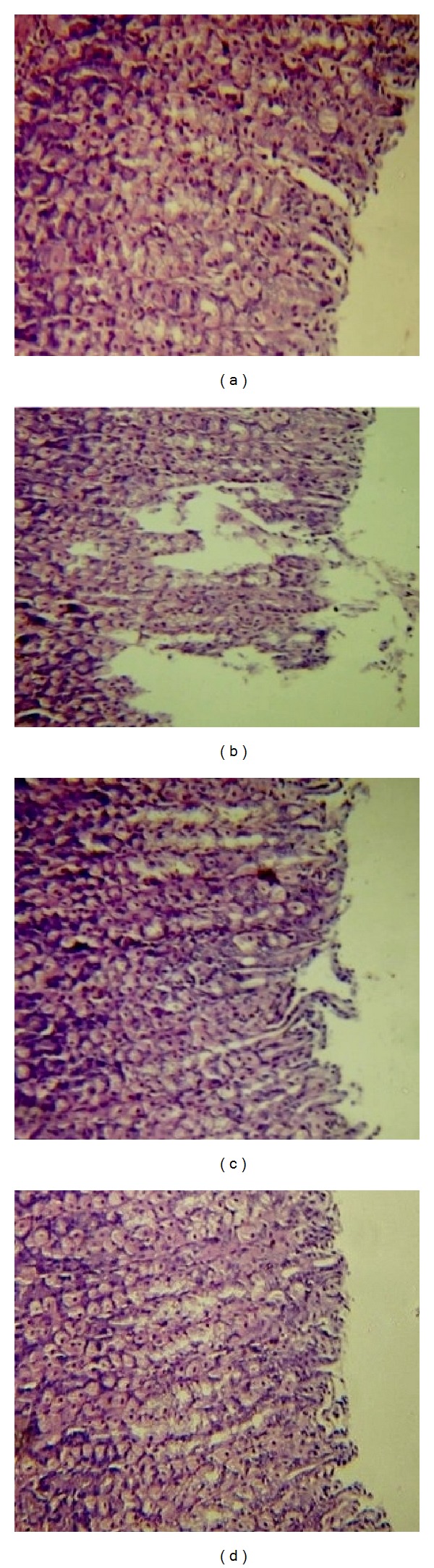
Light micrographs of control (a) rat stomach demonstrating intact stomach mucosa Haematoxylin-Eosin (H&E ×100). Indomethacin -treated group (b), focusing on superficial mucosal cell, had sloughed off gastric mucus, with infiltration of inflammatory cells (H&E ×100). AGE 100 + IN-treated group (c), showing notable healing effect, showing almost normal mucosa, with exception of mild inflammation (H&E ×100). AGE 200 + IN-treated group (d), showing normal mucosa reflecting the healing impact (H&E ×100).

Indomethacin intake induced severe and extensive macroscopic gastric mucosal damage in the irrigated starved rats, characterized by injury in the epithelial layer of the mucosa and sloughed off gastric mucus (Figure 4(b)) as compared to the normal feature in control group (Figure 4(a)). The disruption in the gastric mucosa partially restored after treatment in IN + AGE 100 group (Figure 4(c)). The gastric mucosal tissues of the IN + AGE 200 treated group (Figure 4(d)) showed almost normal and continuous mucosal layer and formation of the epithelial layer. The efficacy of IN + AGE 200 was better than that of misoprostol as revealed in IN + AGE 100 from Figures 4(d) and 4(c), respectively.

### 3.4. Effect of AGE on TNF-*α* Level

**Figure 5:**
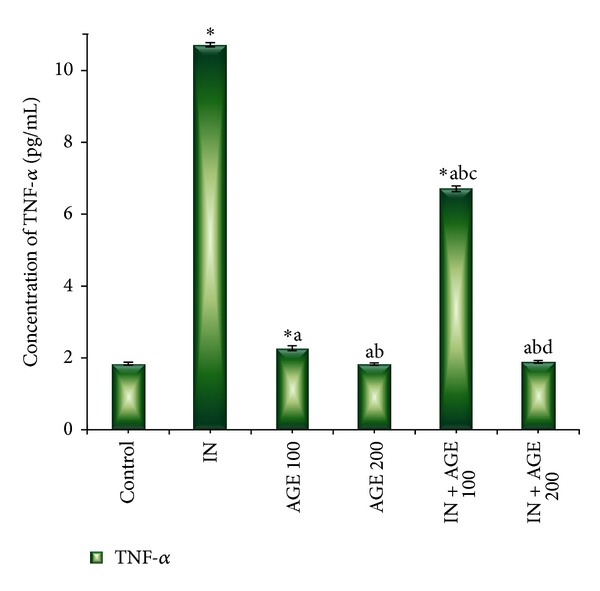
Effect of aged garlic extract (100 or 200 mg/kg) treatments on serum tumor necrosis factor-alpha (TNF-*α*) (pg/mL) levels from rats treated with or without indomethacin. Data expressed as mean ± SE (*n* = 8). Significance (*P* > 0.05) between groups represented by superscripts as (∗), significant as compared to control. (a) Significant as compared to IN-group. (b) Significant as compared to AGE 100 group.-(c) Significant as compared to AGE 200 group. (d) Significant as compared to IN + AGE 100 group.

Serum level of proinflammatory cytokine (TNF*α*) expressed in pg/mL in ulcerated rats upon administration of AGE is presented in Figure 5. Compared with control group the serum level of TNF-*α* (1.84 ± 0.04) significantly increased in indomethacin (10.73 ± 0.06), AGE 100 (2.28 ± 0.07), and IN + AGE 100 (6.73 ± 0.08) groups. Meanwhile, the serum level of TNF-*α* significantly decreased in IN + AGE 100 (1.89 ± 0.04) as compared to the three aforementioned groups.

### 3.5. Effect of AGE on MDA Level

**Figure 6:**
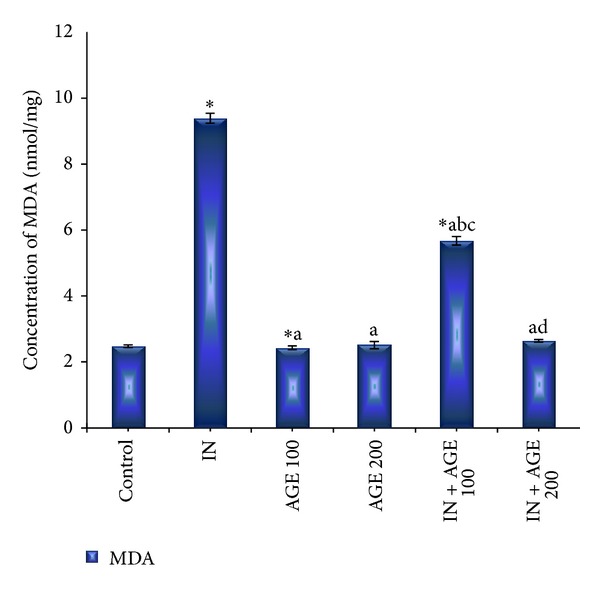
Effect of aged garlic extract (100 or 200 mg/kg) treatments on stomach tissue malondialdehyde (MDA) (nmol/mg) contents from rats treated with or without indomethacin. Data expressed as mean ± SE (*n* = 8). Significance (*P* > 0.05) between groups represented by superscripts as (∗) significant as compared to control. (a) Significant as compared to IN-group. (b) Significant as compared to AGE 100 group. (c) Significant as compared to AGE 200 group. (d) Significant as compared to IN + AGE 100 group.

As represented in Figure 6, administration of indomethacin significantly elevated the gastric mucosal MDA value to 9.48 ± 0.04 nmol/mg as compared to 2.48 ± 0.04 nmol/mg wet tissue for control group. Treatment with AGE to indomethacin-administered group produced significant reduction in gastric mucosal MDA concentration recording 2.43 ± 0.13 nmol/mg IN + AGE 100 and 2.51 ± 0.04 nmol/mg in IN + AGE 200 as compared to indomethacin group.

### 3.6. Effect of AGE on tGSH Level

**Figure 7:**
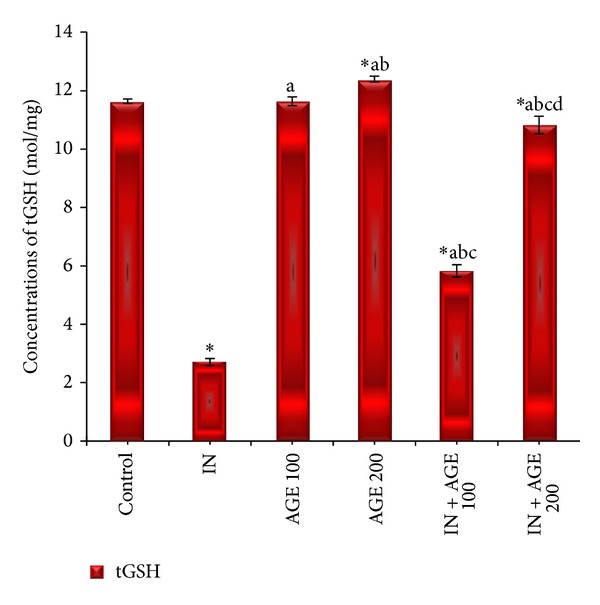
Effect of aged garlic extract (100 or 200 mg/kg) treatments on stomach tissue total glutathione (tGSH) (mol/mg) contents from rats treated with or without indomethacin. Data expressed as mean ± SE (*n* = 8). Significance (*P* > 0.05) between groups represented by superscripts as (∗), significant as compared to control. (a)Significant as compared to IN-group. (b) Significant as compared to AGE 100 group. (c) Significant as compared to AGE 200 group. (d) Significant as compared to IN + AGE 100 group.

As represented in Figure 7, administration of indomethacin significantly decreased the gastric mucosal tGSH value to 2.71 ± 0.12 mol/mg as compared to 11.68 ± 0.08 mol/mg wet tissue for control group. Treatment with AGE to indomethacin-administered group produced significant increase in gastric mucosal tGSH concentration recording 5.83 ± 0.21 mol/mg in IN + AGE 100 group and approaching normal recording 10.85 ± 0.30 mol/mg in IN + AGE 200 as compared to indomethacin group.

### 3.7. Effect of AGE on SOD and CAT Enzyme Activities

**Figure 8:**
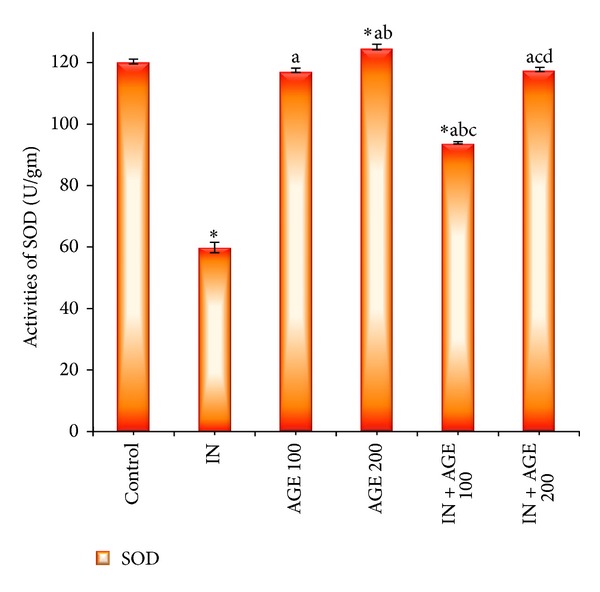
Effect of aged garlic extract (100 or 200 mg/kg) treatments on stomach tissue superoxide dismutase (SOD) (U/mg) activities from rats treated with or without indomethacin. Data expressed as mean ± SE (*n* = 8). Significance (*P* > 0.05) between groups represented by superscripts as (∗), significant as compared to control. (a) Significant as compared to IN-group. (b)Significant as compared to AGE 100 group. (c) Significant as compared to AGE 200 group. (d) Significant as compared to IN + AGE 100 group.

**Figure 9:**
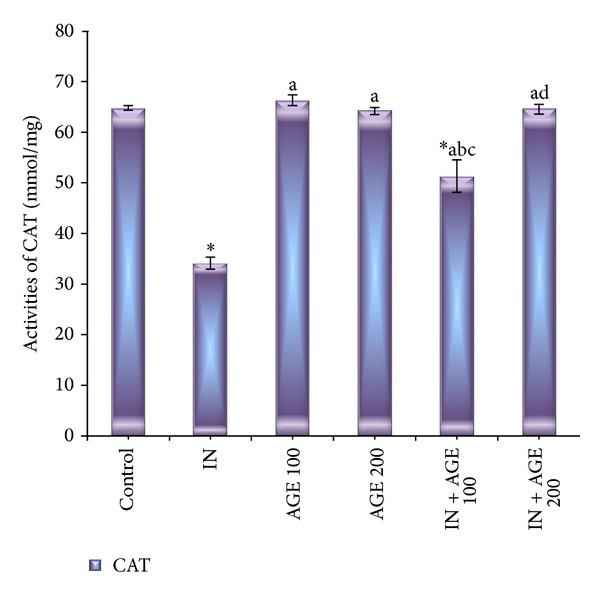
Effect of aged garlic extract (100 or 200 mg/kg) treatments on stomach tissue catalase enzyme (CAT) (mmol/mg) activities from rats treated with or without indomethacin. Data expressed as mean ± SE (*n* = 8). Significance (*P* > 0.05) between groups represented by superscripts as (∗) significant as compared to control. (a) Significant as compared to IN-group. (b) Significant as compared to AGE 100 group. (c) Significant as compared to AGE 200 group. (d) Significant as compared to IN + AGE 100 group.

Figures 8 and 9 represented that administration of indomethacin significantly decreased the gastric mucosal SOD and CAT activities to 59.88 ± 1.72 U/mg and 34.14 ± 1.20 mmol/mg, respectively, as compared to 120.38 ± 0.73 U/mg and 64.81 ± 0.46 mmol/mg tissue for control group, respectively. Treatment with AGE to indomethacin-administered group significantly increased the tissue SOD enzyme activity recording 93.75 ± 0.37 U/mg IN + AGE 100 and approaching the normal activity to reach 117.88 ± 0.69 U/mg in IN + AGE 200 as compared to indomethacin group. In the same manner AGE, coadministration to indomethacin-administered group significantly increased the tissue CAT enzyme activity recording 51.51 ± 3.22 mmol/mg IN + AGE 100 and tending to normalize the activity to reach 64.66 ± 0.96 mmol/mg in IN + AGE 200 as compared to indomethacin group.

### 3.8. Effect of AGE on MPO Enzyme Activity

**Figure 10:**
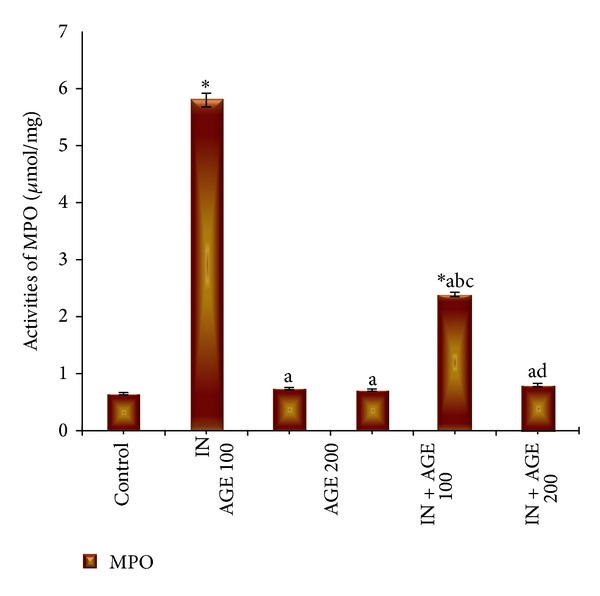
Effect of aged garlic extract (100 or 200 mg/kg) treatments on stomach tissue myeloperoxidase (MPO) (*μ*mol/mg) activities from rats treated with or without indomethacin. Data expressed as mean ± SE (*n* = 8). Significance (*P* > 0.05) between groups represented by superscripts as (∗), significant as compared to control. (a) Significant as compared to IN-group. (b) Significant as compared to AGE 100 group. (c) Significant as compared to AGE 200 group. (d) Significant as compared to IN + AGE 100 group.

The index of neutrophil infiltration in gastric damage by myeloperoxidase enzyme activity was measured in the stomach tissue and represented in Figure 10. In indomethacin treated group MPO activity significantly increased to 5.80 ± 0.12 *μ*mol/mg as compared to 0.65 ± 0.02 *μ*mol/mg tissue for control group. Treatment with AGE to indomethacin-administered group significantly decreased the tissue MPO enzyme activity to reach 2.39 ± 0.04 *μ*mol/mg in IN + AGE 100 group and normalize the activity to reach 0.71 ± 0.02 *μ*mol/mg in IN + AGE 200 as compared to indomethacin group.

## 4. Discussion

There are many factors implicated in the pathophysiology of the indomethacin ulcerogenic potential establishing it as the first-choice drug to produce an experimental ulcer model because of having a higher ulcerogenic potential than other nonsteroidal anti-inflammatory drugs (NSAIDs) [30]. The ulcerogenic mechanism of indomethacin suggested as accompanied with severe oxidative stress in gastric tissue causing damages to key biomolecules such as lipids, proteins, and DNA leading to increased accumulation of MDA, MPO, and accumulation of reactive products altering enzymatic and nonenzymatic antioxidant parameters leading to enhanced oxidative damage during stomach ulceration [15, 31–34]. In addition, the tumor necrosis factor-a (TNF*α*) might be the key signal for NSAID-induced gastric inflammation. Where neutrophil accumulation within the gastric microcirculation and the levels of TNF*α* in the plasma of rats significantly increased following the administration of indomethacin accompanied by gastric injury [12, 35, 36]. In the present study, there are marked damage to the gastric mucosa as evident by macroscopic and histopathological examinations associated with the reduced activities of SOD and CAT and tGSH level, in addition to the elevated TNF*α* level and MPO activity following indomethacin administration.

As regards to the predicted significant elevation of microflora in our ulcer model could be explained through the previous studies where, in experimental models, bacteria colonization at the stomach ulcer site appears to play an important role in exacerbating mucosal injury and has a clear detrimental effect on its healing [37]. Gram-negative bacteria are likely to be responsible for the observed delay in ulcer healing, whereas Gram-positive bacteria may actually promote ulcer healing. Previous study suggested that bacteria other than* Helicobacter pylori* have the capacity to significantly influence the ulcer and that ulcers represent an environment conducive to bacterial growth [38]. Other studies evidenced that persistent colonization of the stomach with* C. albicans* could be achieved in rats by NSAID treatments and that, despite a marked reduction in gastric acid secretion, this infection delays ulcer healing and is accompanied by a fall in mucosal microcirculation in the ulcer area. The delay in ulcer healing induced by* Candida* combined may be associated with gastric mucosal inflammation that involved overexpression and subsequent release of TNF*α* [23, 39].

The current study investigated the anti-inflammatory activity of AGE in two doses of AGE (100 and 200 mg/kg) on indomethacin subjected rats. The experimental results showed the advantage of healing potential of the high AGE dose (200 mg/kg) compared to that at dose 100 mg/kg in the gastric mucosal injury induced by indomethacin. The anti-inflammatory activity of AGE was exhibited through its antioxidant activity by resolving the oxidative stress in gastric tissue. AGE is prepared by soaking garlic in ethanol-water mixture for 20 months, which removes irritant compounds from garlic and solubilizes some of the insoluble compounds. The process converts unstable compounds, such as allicin, to stable substances and produces high levels of water-soluble organosulfur compounds that are powerful antioxidants. These include S-allylcysteine (SAC), AGE's major component, and S-allylmercaptocysteine, unique to AGE. Among other compounds present are low amounts of oil-soluble organosulfur compounds, flavonoids, a phenol, allixin, selenium, and saponins [6, 40–42]. AGE phenolic contents may exert the scavenging activities by donating a hydrogen atom from their phenolic hydroxyl groups [41, 42]. In addition, Na-(1-deoxy-D-fructos-1-yl)-L-arginine (Fru-Arg) was identified as a major antioxidant compound in AGE. The hydrogen peroxide scavenging activity of Fru-Arg was comparable to that of ascorbic acid, suggesting that it could contribute to the pharmacologic effects of AGE through its antioxidant properties [43]. Previously AGE proposed greater safety and efficacy than raw garlic as a therapeutic agent [42]. As regards to the antimicrobial activity of AGE, results in our study are in line with the previous studies, which found that the garlic-derived compound S-allylcysteine (SAC) inhibited the growth of* Escherichia coli* and enhanced the antibiotic effect of gentamycin [44]. Previous study revealed the dose-dependent antimicrobial efficacy of aqueous garlic extract against 133 multidrug-resistant Gram-positive and Gram-negative bacterial isolates and against 10* Candida* spp. The antimicrobial potency of garlic attributed to its ability to inhibit toxin production and expression of enzymes for pathogenesis [45]. The antibacterial and antifungal activities of AGE constituents, allicin, and SAC were previously demonstrated [46–49]. The pattern of resolving oxidative stress in gastric tissue observed through the direct relationship between gastric non-enzymatic tGSH levels and ulcer severity. The tissue, tGSH, and GSH-related enzymes accepted as important protective agents due to their antioxidant properties prevent tissue damage by keeping the ROS at low levels [50, 51]. Aged garlic extract and SAC in both vivo and cell culture-based previous studies recorded to preserve the levels of glutathione peroxidase and glutathione reductase, where glutathione reductase involved in conversion of oxidized glutathione to glutathione [52, 53]. Aged garlic extract treatment significantly prevented induced stress degeneration in morphology and reversed the increased level of MDA and the decreased GSH contents to control values of gastrointestinal mucosa due to its potent free radical scavenging and antioxidant properties [54]. The antioxidant properties of AGE ameliorated oxidative organ injury due to naphthalene toxicity by reversing significantly the elevated MDA levels and MPO activity levels [55]. The study reported that the antioxidant enzymes activities of SOD and CAT have been reversed to normal with 200 mg/kg AGE treatment in IN-administered group these results agree with previous studies reported that garlic extract induces antioxidant effects on rats [56, 57] and suggested that AGE is able to directly scavenge superoxide radicals [58]. Garlic allicin inhibited the TNF-alpha secretion assessing the anti-inflammatory effect of allicin on intestinal epithelial cells [59] and SAC exhibited a dose-dependent inhibition of NF-kappa B activation induced by both TNF-alpha and H_2_O_2_ in human T lymphocytes (Jurkat cells) [60].

In conclusion, the healing activity of AGE at high dose (200 mg/kg) may be resulted from its ability to scavenge ROS produced by indomethacin administration that initiate lipid peroxidation. The mechanism of gastroprotective effects of the AGE on gastric damage induced by indomethacin may be related to its anti-inflammatory actions and its antioxidant properties, which reduce MDA levels and MPO activity and increase tGSH, SOD, and CAT activities. Therefore, our study suggest that AGE was safe and could be a promising new drug for the prevention of NSAIDs-induced gastric damage.
